# The Platform Vector Gene Therapies Project: Increasing the Efficiency of Adeno-Associated Virus Gene Therapy Clinical Trial Startup

**DOI:** 10.1089/hum.2020.259

**Published:** 2020-10-16

**Authors:** Philip J. Brooks, Elizabeth A. Ottinger, Deanna Portero, Richa Madan Lomash, Asaf Alimardanov, Pramod Terse, Xin Xu, Randy J. Chandler, Janelle Geist Hauserman, Eric Esposito, Carsten G. Bönnemann, Charles P. Venditti, Christopher P. Austin, Anne Pariser, Donald C. Lo

**Affiliations:** ^1^ Office of Rare Disease Research,; ^2^ Therapeutic Development Branch, and; ^3^ Organic Acid Research Section, Medical Genomics and Metabolic Genetics Branch, National Human Genome Research Institute (NHGRI);; ^4^ Neuromuscular and Neurogenetic Disorders of Childhood Section, National Institute of Neurological Disorders and Stroke (NINDS); National Institutes of Health, Bethesda, Maryland, USA.; ^5^ Office of the Director, National Center for Advancing Translational Sciences (NCATS);

## INTRODUCTION

There are an estimated 7,000–10,000 different rare diseases that impact the human population.^1^ Around 85% of rare diseases (∼6,500 diseases) are thought to be monogenic disorders, which are diseases caused by mutations in a single gene, with this number increasing by about 200 newly identified diseases each year.^2^ With recent advances in medical science and biotechnology, and several recent approvals for gene therapies and hundreds more in clinical development, highly efficacious disease-modifying therapies and, potentially, cures for most rare monogenic diseases are becoming a real possibility. However, given that only about 5% of rare diseases currently have a specific regulatorily approved treatment, at the current pace of only three to five rare diseases having a first specific treatment approved each year, some 2,000 years would be required for specific treatments for all rare monogenetic disorders to be developed. In fact, many rare monogenetic diseases might never have potentially curative therapeutics approved despite promising therapeutic opportunities, because the traditional one-disease-at-a-time model of commercial therapeutic research and development is too inefficient to justify developing therapeutics for population sizes below a certain threshold.

Because so many rare monogenic diseases are life-limiting conditions, efficacious therapies are urgently needed now. To meaningfully change the current trajectory of rare disease therapeutics development, what is needed are not incremental one-disease-at-a-time approaches, but rather a fundamentally different many-diseases-at-a-time approach that focuses on biological and modality-relevant commonalities across different diseases.

For monogenic diseases caused by recessive mutations leading to a loss of function, there is, at least in principle, a clear therapeutic strategy. Specifically, introducing a working copy of the defective gene (with appropriate regulatory elements) into the clinically relevant cell type(s), at the right time in development and disease progression, should result in either an amelioration, cure, or even prevention of the disease. For many years, the limiting factor in gene therapy has been the ability to effectively deliver therapeutic genes into target cells.

However, for some cell and tissue types, this roadblock has largely been overcome by adeno-associated virus (AAV) vectors. Studies in mice, nonhuman primates, and humans have shown that AAV vectors can effectively deliver therapeutic genes to target cells in muscle, liver, and brain, among other target organs. The U.S. Food and Drug Administration (FDA) has now approved gene therapy products for treating an RPE65 mutation-associated retinal disease, Leber congenital amaurosis,^3^ and a neurodegenerative disease, spinal muscular atrophy.^4^

AAV vectors have been administered in an estimated 250–300 clinical trials and have had a good overall safety record to date in human patients.^5^ However, the results of a study using an AAV vector in children with X-linked myotubular myopathy (XMTM) should be noted. Although early results continue to indicate a clear clinical benefit after gene therapy, the recent reports of the deaths of three boys in the high-dose group in this trial raise obvious safety concerns. Although more details surrounding the clinical events and laboratory parameters will need to be investigated, it has been suggested that the deaths were influenced by a combination of a high total vector dose (which was based on total body weight), as well as an interaction with pre-existing cholestatic liver disease, a recognized hepatic manifestation of XMTM, which was irreversibly exacerbated after the high vector load to the liver.^6^ Even if a predisposing risk factor partly underlies the lethal outcome experienced by these boys, the spectrum of side effects noted in the XMTM trial illustrate the limitations of our current knowledge in being able to predict adverse effects of AAV gene therapy across rare diseases.

AAV vectors are intrinsically disease-agnostic in the sense that their applicability for a particular disease is governed more by their biodistribution as a function of capsid serotype, route of administration and dose, the genetic mechanism to address (for instance, loss of function versus gain of function), and the expression cassette used, rather than by pathophysiological specifics of the disease under consideration. Thus, by putting different therapeutic genes within a chosen AAV capsid, different AAV-mediated gene therapies can be produced to treat different diseases. This modularity of function facilitates the swapping of transgenes, selection of regulatory elements (enhancer, promoter, *etc.*), and alterations of the capsid, which allow different tissues and cell types to be targeted. As such, AAV as a vector is fundamentally a platform modality^5^: a programmable multipurpose vehicle that can be used to deliver a variety of different therapeutic payloads to disease-relevant cells. From this we can also hypothesize that the more similar a group of diseases are in terms of their target cells and genetic mechanism, the more streamlined the modular approach can be conceived of.

In contrast to the inherent platform potential of AAV vectors, AAV clinical development to date has not prioritized maximizing the efficiency of the AAV platform. This one-disease-at-a-time approach to clinical development does not fully leverage the platform capacity of AAV vectors, capitalize on any commonalities in preclinical development, or promote sharing of knowledge across individual therapeutic programs. This results in duplication of effort, as well as suboptimal use of time, funding, animals, and other scarce resources.

An important consideration for choosing diseases for commercial therapeutics development is disease prevalence, which results in a focus on diseases that affect a relatively larger numbers of patients and thus present larger market sizes. However, roughly 80% of rare diseases affect <1 person per million^7^ and this very low prevalence serves to limit commercial attention to developing treatments for the majority of rare monogenic diseases.

This lack of commercial viability of many rare disease therapeutics is exacerbated by the fact that gene therapies, in contrast to small molecule drugs and antisense oligonucleotides, are designed to be one-time treatments. This means that patients who participate in gene therapy clinical trials cannot also be consumers of these therapies should they receive marketing approval.

Collectively, the constraints of the current development model present formidable challenges to the creation of scientifically practical and medically needed gene replacement therapies for rare diseases. This is an acute problem because the scientific rationale and clinical impetus for the application of gene therapy to monogenic diseases is independent of disease prevalence. As such, therapies for numerous very low prevalence monogenic disorders are currently scientifically feasible but cannot move forward due to operational and financial considerations. The biggest hurdle remains traversing the path between proof-of-concept (POC) data in an animal model to the opening of a clinical trial. In the absence of commercial interest, the costs and burden of developing gene therapies for noncommercially viable diseases often fall to patients and their families, who are usually not prepared for such a responsibility, either in terms of scientific training, regulatory experience, or access to financial support. This reality also has the unfortunate effect of resulting in disparities in access to gene therapy trials based not on scientific considerations, but socioeconomic and other factors.

To address this operational problem, the U.S. National Institutes of Health (NIH) National Center for Advancing Translational Sciences (NCATS), in collaboration with the National Institutes of Neurological Disorders and Stroke (NINDS) and the National Human Genome Research Institute (NHGRI), have initiated the Platform Vector Gene Therapy (PaVe-GT) pilot project. PaVe-GT seeks to increase the efficiency of clinical therapeutics development and trial startup by multiplexing preclinical and clinical development and freely disseminating the information gained on the PaVe-GT website: https://pave-gt.ncats.nih.gov/. PaVe-GT is an experimental translational science initiative that aims to leverage the power of platform vectors and disease relatedness to help deliver on the promise of gene therapy to patients with rare diseases of no commercial interest.

## WHAT IS PaVe-GT?

**Figure 1.**
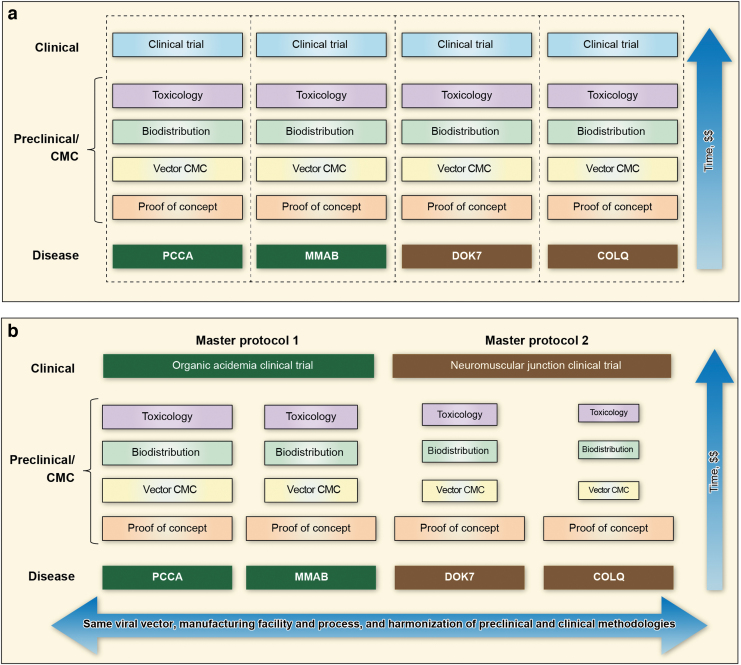
PaVe-GT development process overview. **(a)** Traditional one-disease-at-a-time approach: The development process for the four diseases/genes under study would proceed from bottom to top through each of the CMC, preclinical, and clinical phases separately with no leveraging of resources from one disease-program to another, and resulting in separate clinical trials. No resource savings would be expected from this approach. **(b)** PaVe-GT many-diseases-at-a-time approach: PaVe-GT seeks to leverage available information from each of the four related programs to others with the hypothesis that time and resources can be saved in the CMC, preclinical, and clinical phases. From bottom to top, expected resource savings (indicated by progressively smaller text boxes) beginning in vector product characterization, biodistribution, and toxicology phases. In the clinical phase, each of the two therapeutic-disease areas for the organic acidemias and NMJ diseases can be grouped into master protocols rather than being performed as separate trials. CMC, chemistry, manufacturing, and controls; NMJ, neuromuscular junction; PaVe-GT, Platform Vector Gene Therapy.

PaVe-GT is a pilot project whose main goal is to test whether the efficiency of gene therapy development and clinical testing can be substantially increased by using a standardized process for four different rare diseases. PaVe-GT will use the same AAV serotype, AAV-9, as a platform vector to develop gene therapy products for all four diseases (Fig. 1). AAV-9 was selected for this project because of its broad cell and tissue tropism, including the liver, muscle, and central nervous system (CNS). The AAV-9 vector used in clinical trials here will be produced in the same manufacturing facility, using the same production and purification methods, with the only difference being the therapeutic gene constructs. Although POC studies will be conducted individually for the selected disorders, we hypothesize that additional efficiencies will be gained in the biodistribution studies and potentially, toxicology, as the preclinical testing advances.

In clinical research, the term platform is used in multiple different ways. Therefore, it is important to clearly distinguish clinical development based on a platform viral vector (as used here) versus a platform clinical trial. A platform clinical trial has generally been defined as a durable infrastructure to support the testing of multiple treatments across one or more types of diseases or patient populations.^8^ By using a platform, patients with a heterogeneous disease (such as cancers) can be treated in parallel or sequentially with different candidate therapeutics under a “master protocol” without the need to reassemble clinical trial infrastructure to test each product, as well as offering the possibility of treatment to a broader cohort of patients.

Master protocols are generally defined as a framework under which multiple drug studies are operated under one overarching protocol.^8^ To date, clinical trial platforms and master protocols have most commonly been used in oncology indications, although they have substantial potential for rare monogenic diseases as well. In PaVe-GT, the main focus is on the benefits resulting from the use of a platform AAV vector. However, PaVe-GT also plans to incorporate clinical trial platform concepts, including the use of master protocols to evaluate the AAV gene therapies in phenotypically related diseases. Specifically, although we are studying four different diseases, we will have two clinical trial protocols; one for the organic acidemias, and one for the congenital myasthenic syndromes (CMS) (Fig. 1b).

The PaVe-GT project was conceived to openly share project results and lessons learned with the research and patient communities in such a way that the information could be useful to any party interested in developing a subsequent gene therapy using an identical or similar approach. Information and results from the program will be made publicly available on the PaVe-GT website in as timely a manner as possible, including POC, toxicology and biodistribution data, Investigational New Drug (IND) filings and communications with the FDA, and other study documents.

We note some limitations to the PaVe-GT approach. For this pilot project, an independent contract manufacturing facility will perform the manufacturing of the AAV vector. NIH will not have access to proprietary aspects of that manufacturing process and, therefore, will not be able to share that information with the public. The PaVe-GT project will attempt to identify an equivalent widely accessible manufacturing process with meaningful freedom to operate. Therefore, it is possible but not guaranteed that future independent gene therapy projects will be able to reuse PaVe-GT data in their INDs. Another limitation relates to the generalizability of the PaVe-GT project management approach and strategies. Although it is anticipated that first-time gene therapy developers in the research and patient communities will be able to learn a great deal from the PaVe-GT model of project management, there may be some elements of project planning and coordination that are not generalizable, because of unique limitations and opportunities specific to the Federal government. However, the POC data and approach, toxicology and biodistribution data, the IND filings and our communications with the FDA that will be made public should benefit many stakeholders interested in AAV gene therapy trials, particularly new groups developing gene therapies for diseases of no commercial interest. In addition, the PaVe-GT approach should also generate valuable data regarding payload-independent safety concerns, which will also be put into the public domain as soon as possible to benefit other AAV clinical trial efforts (see Morales *et al.*^9^).

## DISEASE SELECTION PROCESS

In principle, almost any monogenic disease that is amenable to AAV-based delivery could have been included in PaVe-GT. The four diseases that will be targeted in PaVe-GT were selected based on practical and budgetary considerations. Since PaVe-GT is a pilot program, by definition, it implies a high degree of programmatic risk so it was decided to conduct the program within the NIH Intramural Research Program (IRP). This allows the program to leverage the unique clinical research resources available at the NIH Clinical Center, facilitate institutional communication, and streamline project startup. Second, diseases that were already under study by investigators within the NIH IRP, and were amenable to AAV gene therapy, were selected. Importantly, the investigators also have long experience with clinical trials in rare diseases, including gene therapies, and have already developed clinical outcome measures based on natural history studies, which can be leveraged for this project. Third, working with investigators who are all Federal employees facilitates the goal of making all data public, by simplifying considerations regarding intellectual property that could have introduced delays and complications. Finally, at the time of disease selection, we were not able to identify any commercial interest in any of diseases under study in PaVe-GT. It is important to emphasize that although the PaVe-GT pilot is limited to four diseases, these were explicitly chosen as demonstration cases and it is our hope that the data and findings will be useful to others developing AAV gene therapies for other rare monogenetic diseases.

## DISEASES UNDER STUDY

The rare monogenic diseases selected include two organic acidemias and two CMS. The organic acidemias under study are propionic acidemia (PA) (caused by propionyl-CoA carboxylase A [PCCA] deficiency) and isolated methylmalonic acidemia (MMA) (cobalamin type B MMA [MMAB] deficiency). The CMS under study are downstream of tyrosine kinase 7 (DOK7) deficiency and collagen Q (COLQ) deficiency.

### Organic acidemias

#### PA caused by PCCA deficiency

**Figure 2.**
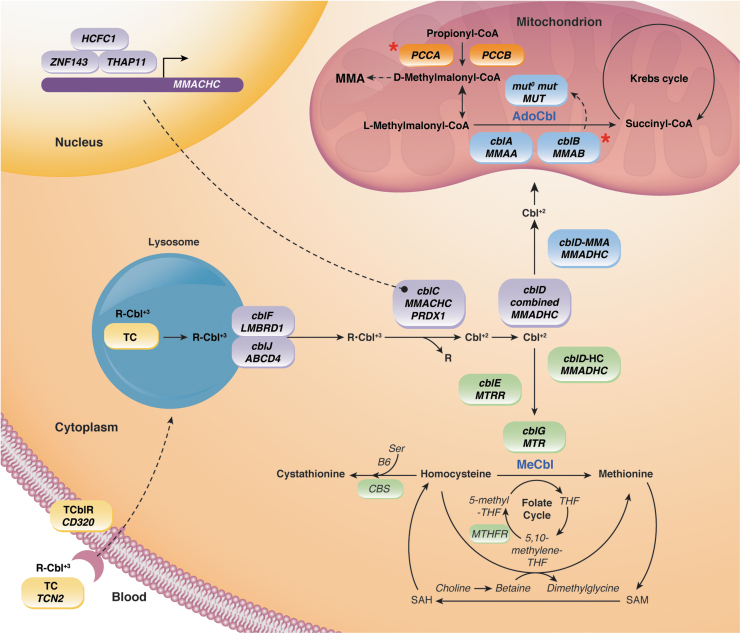
Propionate and cobalamin metabolism. The organic acidemias under study in PaVe-GT are PA caused by mitochondrial-localized PCCA deficiency,^7^ an enzyme responsible for conversion of propionyl-CoA to d-methylmalonyl-CoA, and isolated MMA MMAB caused by cobalamin B deficiency^14^ (PCCA and MMAB indicated by *red asterisks*). PCC deficiency (caused by PCCA or PCCB deficiency, both of which occur with equal frequencies) results in elevated levels of toxic propionyl-CoA derived from metabolism of some amino acids (isoleucine, valine, threonine, and methionine), propionate from gut flora, cholesterol and odd-chain fatty acids, which contribute to the production of propionyl-CoA. MMAB is one of several enzymes involved in the conversion of vitamin B12 to 5′-deoxyadenosylcobalamin, the active cofactor required by the mitochondrial enzyme MMUT to function in the conversion of l-methylmalonyl-CoA to succinyl-CoA. Isolated MMA due to enzyme or cofactor deficiencies lead to elevations in methylmalonic acid. Similar to PA, patients with MMA are unable to metabolize certain amino acids and lipids. MMA, methylmalonic acidemia; MMAB, cobalamin type B MMA; MMUT, methylmalonyl-CoA mutase; PA, propionic acidemia; PCC, propionyl-CoA carboxylase; PCCA, propionyl-CoA carboxylase A; PCCB, propionyl-CoA carboxylase B.

PA is a rare inherited autosomal recessive metabolic disorder caused by a deficiency in propionyl-CoA carboxylase (PCC) activity^10^ (Fig. 2). Patients diagnosed with PA typically present in the early newborn period with a metabolic crisis, which may manifest as vomiting, seizures, lethargy, hypotonia, and encephalopathy. These symptoms may be recurrent, with episodes triggered by feeding or infection, and can result in permanent neurological damage and may be fatal if not promptly recognized and treated.

The current management of PA relies upon dietary restriction of branch chain amino acid precursors, carnitine supplementation, and aggressive management during episodes of intercurrent infections and stress. Despite vigilant monitoring and proactive medical management, patients can suffer from metabolic decompensations, hyperammonemia, pancreatitis, cardiomyopathy, sudden death from ventricular arrhythmia, strokes of the basal ganglia, poor growth, cytopenias, and renal disease.^11^ The severe disease burden, high rates of morbidity and mortality, and poor quality of life experienced by PA patients have led to the use of elective liver transplantation as a surgical treatment for PA, to restore PCC activity and ameliorate or eliminate disease-related symptoms. The use of expanded newborn screening to detect PA patients in the neonatal period adds another dimension of urgency to the development of new therapies for PA. AAV gene therapy is a potentially promising new therapy for which preclinical studies using various murine models of PA caused by PCCA deficiency have demonstrated a therapeutic benefit.^12,13^ In addition, a natural history study of PA is underway at the NIH (https://clinicaltrials.gov/ct2/show/NCT02890342).

#### MMA (caused by MMAB deficiency)

Isolated MMA is a group of heterogenous metabolic disorder most commonly caused by complete or partial deficiency of the mitochondrial enzyme methylmalonyl-CoA mutase or a defect in the transport or synthesis of its cofactor, 5′-deoxyadenosylcobalamin^14^ (Fig. 2). The cobalamin B type of MMA is caused by mutations in the *MMAB* gene and it is one of the rarest forms of MMA. The cobalamin B type of MMA is inherited in an autosomal recessive manner and can be detected by newborn screening, and then confirmed by genetic testing. Patients with the cobalamin B type of MMA experience severe symptoms, which can appear in early infancy or the first year of life, of acid–base imbalance, high levels of ammonia, lethargy, vomiting, dehydration, hypotonia, and delayed development. Long-term complications include kidney disease, pancreatitis, cytopenias, and neurological damage from metabolic strokes of the basal ganglia.

There is no cure for the cobalamin B type of MMA, but treatments include dietary restriction of protein, vitamin B12 given as an injection, carnitine, and intermittent antibiotics. Despite medical and dietary management, these patients can experience significant medical complications that underscore the need for new therapies. Preclinical AAV gene therapy has been used successfully to treat mouse models of MMA^15^ caused by mutations in a related gene (*Mmut*), and by extension, is predicted to be equally effective for the treatment of the cobalamin B type of MMA. The NIH currently is conducting a natural history study of MMA, which has enrolled >200 participants (https://clinicaltrials.gov/ct2/show/NCT00078078).

It is anticipated that the platform design of PaVe-GT will also be beneficial for other forms of PA and MMA, given the nature of the enzyme defects and metabolic pathway, and if successful, may enable a master treatment protocol.

### Congenital myasthenic syndromes

**Figure 3.**
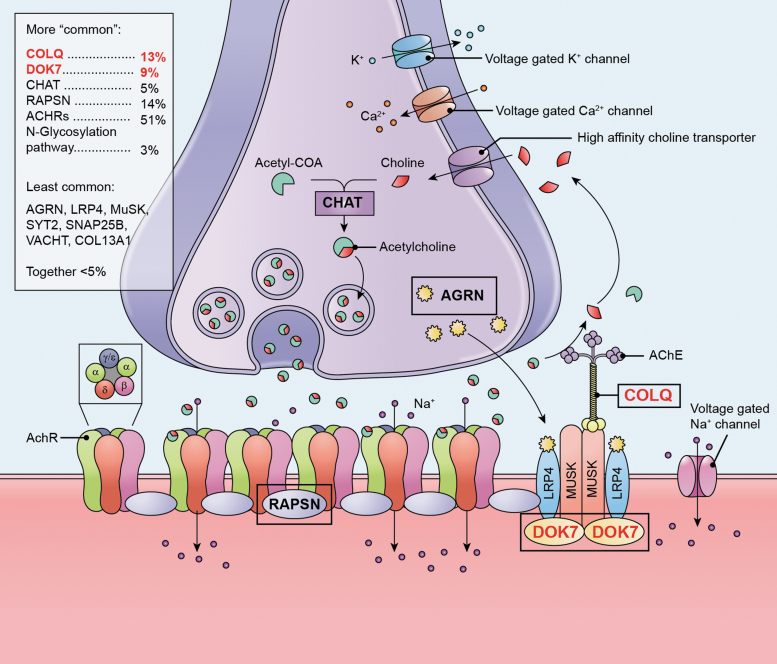
Molecular components of the NMJ affected in CMS. CMS are a collection of diseases caused by pathogenic genetic variants in the molecular components of the NMJ (adapted from ^18^ and ^23^). Defects in all areas of the NMJ—including the presynaptic cell (nerve), synaptic cleft and postsynaptic cell (muscle)—have been causatively linked to the development of CMS, with several of these genes indicated in the figure (*within black boxes*; distribution of genes causatively linked to CMS *within box*^18^). The two forms of CMS chosen for the PaVe-GT initiative are DOK7 and COLQ deficiencies (appear in *red text* within *black boxes*). DOK7 functions primarily to activate MuSK, which allows for the subsequent clustering of acetylcholine receptors in postsynaptic muscle cells.^19^ COLQ is a specific nonfibrillar collagen, which anchors AChE in the basal lamina of the mammalian NMJ.^22^ Without proper function these proteins in patients with CMS, NMJ signaling is altered. AChE, acetylcholine esterase; CMS, congenital myasthenic syndromes; COLQ, collagen Q; DOK7, downstream of tyrosine kinase 7; MuSK, muscle-specific tyrosine kinase.

CMS are a group of inherited disorders in which there is impairment of the neuromuscular junction (NMJ) caused by pathogenic genetic variants in many of the molecular components that make up the NMJ^16,17^ (Fig. 3). Although dominantly acting forms exist, most types are inherited in an autosomal recessive pattern with early onset and loss of function as the mechanism, making them amendable to gene replacement approaches. The NMJ as a specialized structure has been extensively investigated; its molecular composition, morphology, and physiology are well understood, including the regulation of NMJ-specific gene expression.

CMS are rare, affecting less than nine in one million children depending on the population.^15^ Although symptoms vary depending on the underlying cause of the disease, they may include muscle and respiratory weakness, eyelid drooping and problems with eye alignment, delayed motor development, excessive activity induced weakness (muscle fatigue), contractures, and skeletal deformities.

Clinical consequences of NMJ dysfunction are quantifiable by a number of outcome measures, making the NMJ a prime candidate to study the clinical benefits of gene therapy. In addition, since these disorders are not primarily degenerative, as nerve and muscle integrity are largely maintained, restoration of function to the neuromuscular unit should be achievable.

#### DOK7 and COLQ deficiency

The two forms of CMS chosen for the PaVe-GT initiative are DOK7 and COLQ deficiency. These two forms account for about 20% of CMS cases combined.^18^ The *DOK7* gene encodes for DOK7 protein, which is essential for postsynaptic specialization of the NMJ.^19,20^ CMS linked to *DOK7* variants is most often inherited recessively and may result from missense, nonsense, splice site, and/or frameshifts mutations. Although many different CMS types share general phenotypic characteristics, DOK7-deficiency patients often present with delayed motor milestones, difficulty walking at disease onset, and some report facial and bulbar weakness. However, fluctuation of weakness and fatigability may be much less prominent compared with other forms of CMS or only apparent over longer periods of time.^21^ Thus, this condition may be mistaken for a fixed myopathy rather than a CMS, which may delay diagnosis. Despite this, no obvious correlation of clinical symptoms and location of mutations with the gene have been established.

COLQ is a specific nonfibrillar collagen encoded by the *COLQ* gene, which anchors acetylcholine esterase (AChE) in the basal lamina of the mammalian NMJ.^22^ One end of COLQ binds to AChE, whereas the opposite end binds to muscle-specific tyrosine kinase at the postsynaptic membrane. Similar to DOK7 deficiency, the earliest symptoms are detected in the neonatal period or during childhood and include muscle hypotonia, fatigue, respiratory insufficiency, and delayed motor development.

Although pyridostigmine is routinely used for acquired myasthenia gravis and recessive *ACHE* mutations that cause CMS, this treatment is not usually effective in CMS resulting from *COLQ* or *DOK7* mutations, and may worsen symptoms.^23^ In addition, there are varied responses to other treatments, such as salbutamol.^24^ Of particular relevance to PaVe-GT, preclinical POC studies have been published supporting the use of AAV gene therapy in a mouse model of DOK7 deficiency,^25^ and a COLQ knockout animal model is available.^26^

## PROJECT MANAGEMENT CHALLENGES AND STRATEGY

Building and implementing processes to bring innovative solutions into practice to solve translational problems is a collaborative effort requiring tightly coordinated teamwork, and is a central “translational science” problem as NCATS defines it.^27^ A key component is the assembly of a cohesive group of people, including scientists, clinicians, and regulators with different areas of expertise such as assay development, *in vitro* and *in vivo* pharmacology, formulation, manufacturing, and toxicology with a shared vision and goals, and most importantly, a willingness to work together and to openly share ideas, critical tools, and data to meet the projects' goals. The critical first step after assembly of the team is establishing the goals of the program, developing an agreed upon research plan that serves as a roadmap for the studies, and outlining resources needed to accomplish the plan, from preclinical to clinical phases, assigning roles and responsibilities for each collaborator, and establishing initial timelines.

This centralized project management will be key in driving the PaVe-GT project forward to its end goals, by ensuring the necessary resources are available, tracking activities and studies, monitoring overall project costs, and fostering the cohesive team environment. For this purpose, NCATS utilizes project managers with relevant scientific expertise in preclinical development. Their role is to understand both the scientific and strategic goals of the project so that they can proactively and effectively coordinate and prioritize the sharing of critical information, track progression of the project milestones, and quickly respond appropriately if roadblocks arise during the implementation of the project. They create a strong collaborative team network, stemming from commitment and passion by every member of the team, who has to be proactive, responsive, and follow through on action items and deliverables on time to support successful execution of the overall strategy. In turn, the team needs to be flexible to address unexpected data results or mitigate problems encountered along the way to be able to adapt and make changes to the project plan. Thus, tight coordination and effective communication among stakeholders are essential for all the “moving parts” in a very complex process to come together to facilitate a successful project outcome, particularly in a multilateral noncommercial setting. As such, PaVe-GT will aim to provide a useful blueprint for managing gene therapy product development and testing, sharing information, best practices, and lessons learned along the way with the public in an open forum that will help others to follow the path and decrease their burden to achieving gene therapy especially for rare diseases with small numbers of patients.

## FDA ENGAGEMENT STRATEGY

Engaging FDA as a research partner is an essential element of the PaVe-GT project design. The NIH PaVe-GT team initially engaged FDA Center for Biologics Evaluation and Research (CBER) for nonbinding discussion of the general PaVe-GT concept, including the acceptability of developing and clinically testing four gene therapies for four diseases in a standardized process, exploring mechanisms for seamless information sharing between gene therapy applications, such as the use of master files, and hearing FDA's concerns regarding maintaining high-quality science in an expedited program.

Given the thousands of rare monogenic disorders, and the largely unmet medical needs of the rare disease patients, as communicated to NIH in these discussions as well as in public meetings, FDA CBER has voiced their support for designing programs that will get more efficacious therapies to more patients quickly without compromising safety, even in very small patient populations.^28^

FDA has also published guidances for gene therapy programs on a variety of topics (https://www.fda.gov/vaccines-blood-biologics/biologics-guidances/cellular-gene-therapy-guidances). Importantly, these guidances urge frequent high-quality communications between gene therapy sponsors and regulators at all steps of the development process starting with early phase INitial Targeted Engagement for Regulatory Advice on CBER producTs (INTERACT) meetings.

An essential aspect of PaVe-GT will be making available, in as near real time as possible, all written communications with FDA. The purpose of this is to inform rare disease stakeholders of questions, responses, and advice we have received, and to provide real examples of FDA interactions, especially for those less experienced in drug development science and processes. From our discussions to date, FDA has indicated that they are highly supportive of our approach and NCATS' plan for information sharing.

## CONCLUSION

The scientific rationale for AAV gene therapy in numerous monogenic diseases is well founded, and the tools and expertise to develop and implement clinical development programs are currently within reach for many of these disorders. However, despite these scientific advances, due mainly to financial considerations, most commercial AAV development efforts will likely focus on the most prevalent rare diseases. Thousands of noncommercially viable rare diseases may thus remain “orphaned” despite the existence of tools with significant potential to meaningful alter the course of these diseases.

Rare monogenic diseases are not, in fact, rare given the large number of diseases and, collectively, the large number of patients affected. Monogenic disorders, by definition, are diseases that one is born with, and disproportionately affect young children. Ameliorating and preventing adverse metabolic and developmental consequences in children born with what should now be considered treatable conditions adds to the sense of urgency for and the need to radically alter the current trajectory of rare disease therapeutics development. NIH is the world's largest public research agency and fostering innovative research strategies to improve health is one of its main goals (https://www.nih.gov/about-nih/what-we-do/mission-goals). The PaVe-GT project is intended to advance beyond the one-disease-at-a-time paradigm typically followed for rare diseases, by testing how much we can increase the efficiency of new gene therapy development and clinical testing by explicitly taking advantage of the platform capacity of AAV vectors with the larger intention of having more gene therapies becoming operationally feasible in the near-term, especially for these very low prevalence disorders. A key aspect of PaVe-GT is to disseminate program results and regulatory documents as broadly as possible, with the ultimate goal of benefiting future gene therapy clinical development programs for diseases of no apparent commercial interest.
